# A Study on the Dose Distributions in Various Materials from an Ir-192 HDR Brachytherapy Source

**DOI:** 10.1371/journal.pone.0044528

**Published:** 2012-09-05

**Authors:** Shih-Ming Hsu, Chin-Hui Wu, Jeng-Hung Lee, Ya-Ju Hsieh, Chun-Yen Yu, Yi-Jen Liao, Li-Cheng Kuo, Ji-An Liang, David Y. C. Huang

**Affiliations:** 1 Department of Biomedical Imaging and Radiological Science, China Medical University Hospital, Taichung, Taiwan, ROC; 2 Department of Engineering and System Science, National Tsing Hua University, Hsinchu, Taiwan, ROC; 3 Health Physics Division, Institute of Nuclear Energy Research, Longtan, Taiwan, ROC; 4 Department of Medical Imaging and Radiological Sciences, Kaohsiung Medical University, Kaohsiung, Taiwan, ROC; 5 Department of Radiation Oncology, China Medical University Hospital, Taichung, Taiwan, ROC; 6 School of Medical Laboratory Science and Biotechnology, Taipei Medical University, Taipei, Taiwan, ROC; 7 Radiation Oncology, Memorial Sloan-Kettering Cancer Center, New York, New York, United States of America; 8 Radiation Oncology, Faculty of Memorial Sloan-Kettering Cancer Center at Rockville Centre, Rockville Centre, New York, United States of America; Institut Gustave Roussy, France

## Abstract

Dose distributions of ^192^Ir HDR brachytherapy in phantoms simulating water, bone, lung tissue, water-lung and bone-lung interfaces using the Monte Carlo codes EGS4, FLUKA and MCNP4C are reported. Experiments were designed to gather point dose measurements to verify the Monte Carlo results using Gafchromic film, radiophotoluminescent glass dosimeter, solid water, bone, and lung phantom. The results for radial dose functions and anisotropy functions in solid water phantom were consistent with previously reported data (Williamson and Li). The radial dose functions in bone were affected more by depth than those in water. Dose differences between homogeneous solid water phantoms and solid water-lung interfaces ranged from 0.6% to 14.4%. The range between homogeneous bone phantoms and bone-lung interfaces was 4.1% to 15.7%. These results support the understanding in dose distribution differences in water, bone, lung, and their interfaces. Our conclusion is that clinical parameters did not provide dose calculation accuracy for different materials, thus suggesting that dose calculation of HDR treatment planning systems should take into account material density to improve overall treatment quality.

## Introduction

High dose rate (HDR) remote afterloading system represents the commonly used brachytherapy equipment in modern hospitals, with ^60^Co and ^137^Cs preceding ^192^Ir as radioactive sources. Clinically, ^192^Ir is used in brachytherapy such as interstitial and intracavitary radiation therapy. An analysis of the ^192^Ir energy spectrum reveals 110 energies ranging from 9 to 885 keV (an average of approximately 370 keV) [1]. Since dose gradients near radioactive sources are very high, it can be difficult to measure dose distributions in the surrounding tissues.

In 1995, the American Association of Physicists in Medicine Task Group 43 (AAPM TG-43) published a report on dose calculations in brachytherapy [1]. The commonly used dose calculation algorithms were based on the assumption of water surrounding the radioactive source. However, our human organs are not homogeneous in density, with important variation noted for the nasopharynx, bronchi, lungs (density much lower than water) and bones (density much higher than water). Dose errors can result, potentially leading to malignant cancer relapses or other serious side effects.

Several researchers have addressed the topic of ^192^Ir dose distributions in materials other than water. Meredith used a cylinder ionization chamber to measure ^192^Ir exposure rates in air and water [2]. No corrections were applied to chamber size, and the largest errors occurred near the source. Meisberger measured ^192^Ir dose rates in air and water using a 3 mm×3 mm scintillation detector, and the reported results were very close to Meredith’s [3]. Nath et al. used a solid water phantom for thermoluminescent dosimeter (TLD) measurement and made comparisons with Monte Carlo (MC) calculations [4]. In addition to radial dose variation they also calculated and measured dose rate constants, and their results matched those from MC calculations for ^192^Ir as well as Meisberger’s data. Nath et al.’s dose calculation equations represent a scaled-down version of the 1995 AAPM TG-43 equation.

Dissatisfaction with one-dimensional source radial dose distribution data motivated experiments involving 2-dimensional (2-D) plane and 3-dimensional (3-D) dose distributions. Researchers found that the multi-dimensional dose distributions could be acquired once the former was determined [5], [6], [7], [8], [9]. Mishra et al. used a 0.147 cm^3^ ionization chamber to measure ^192^Ir anisotropy function in a water phantom [10]. Their measurements were compared with other studies, and concluded that their TLD and ionization chamber results were within acceptable levels of measurement uncertainty when detector volume was considered. Karaiskos et al. also published their MC calculations of ^192^Ir dose parameters in water and compared them with actual dose distribution measurements [5]. Their results (presented as the *g(r)* radial dose function and *F(r,*θ*)* anisotropy function) were in general agreement with those reported from other studies.

**Figure 1:**
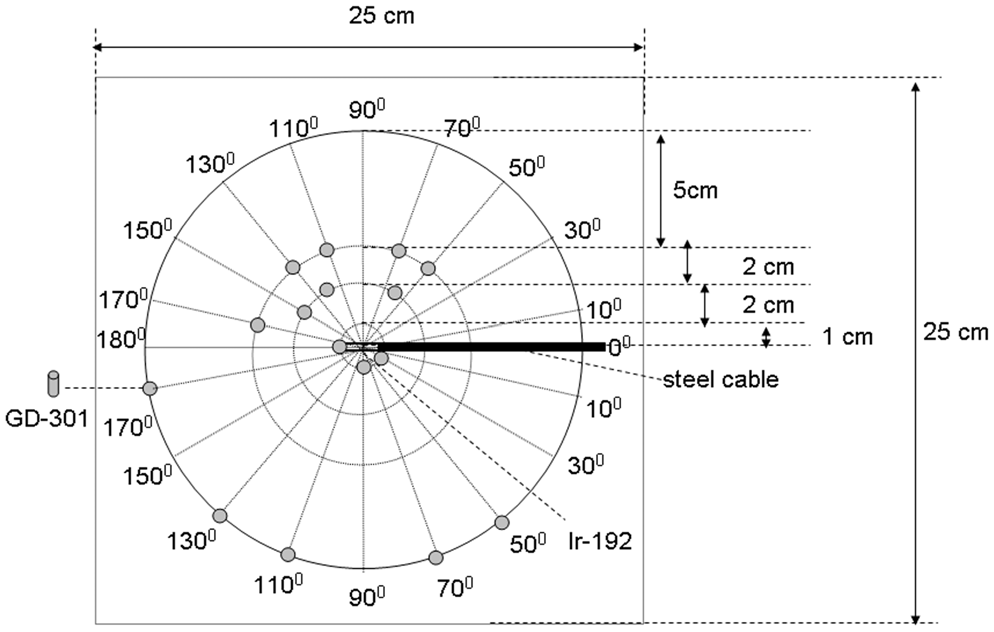
Radiophotoluminescent glass dosimeter (GD-301) measurement position.

### Gafchromic Film Measurements

Accordingly, researchers are increasingly using MC codes to calculate ^192^Ir dose distributions [5], [6]. Most dose calculations are still based on assumptions of water surrounding the radioactive source [3], [5], [6], [8], [9], [11], thus the motivation behind additional researches were on ^192^Ir dose distributions in lung tissue or bone. Kassas et al. also used MC code to simulate dose modification factors for ^192^Ir HDR irradiation [12]. Kassas demonstrated the importance of ensuring the minimum distance required between the balloon’s surface and the breast’s interface with bone, lung, and skin. Pantelis et al. reported that skin and lung dose calculations by the treatment planning system could be thought of as a conservative approach to the overestimated dose reports [13].

In this study, we investigated the dose distributions in various materials from an Ir-192 HDR brachytherapy source. Three MC codes EGS4, FLUKA, and MCNP4C were used to calculate ^192^Ir dose distributions in water, lung, bone, and their interfaces. Results were verified by using radiophotoluminescent glass dosimeter (RPLGD) Gafchromic film measurements.

**Figure 2:**
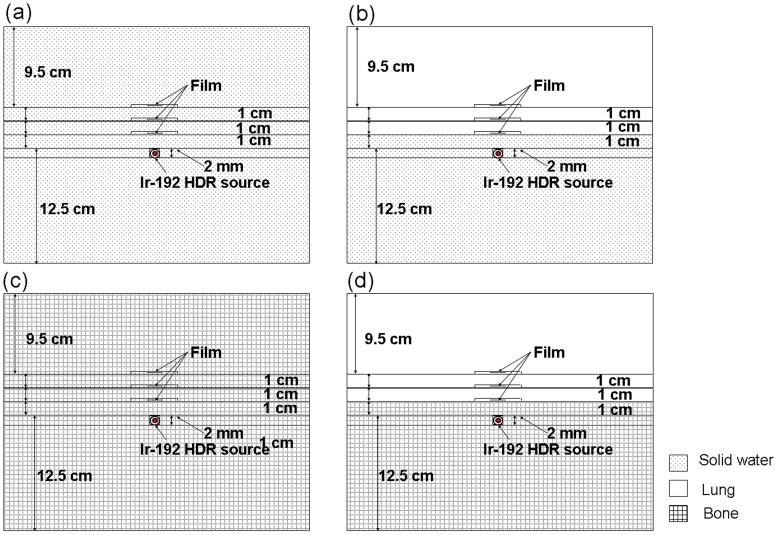
Gafchromic film measurement procedure. Source and film are surrounded by a 25×25×25 cm^3^ phantom. Shown are set-ups for measuring (a) homogeneous solid water phantoms, (b) solid water-lung phantom interfaces, (c) bone phantoms, and (d) bone-lung phantom interfaces.

**Figure 3:**
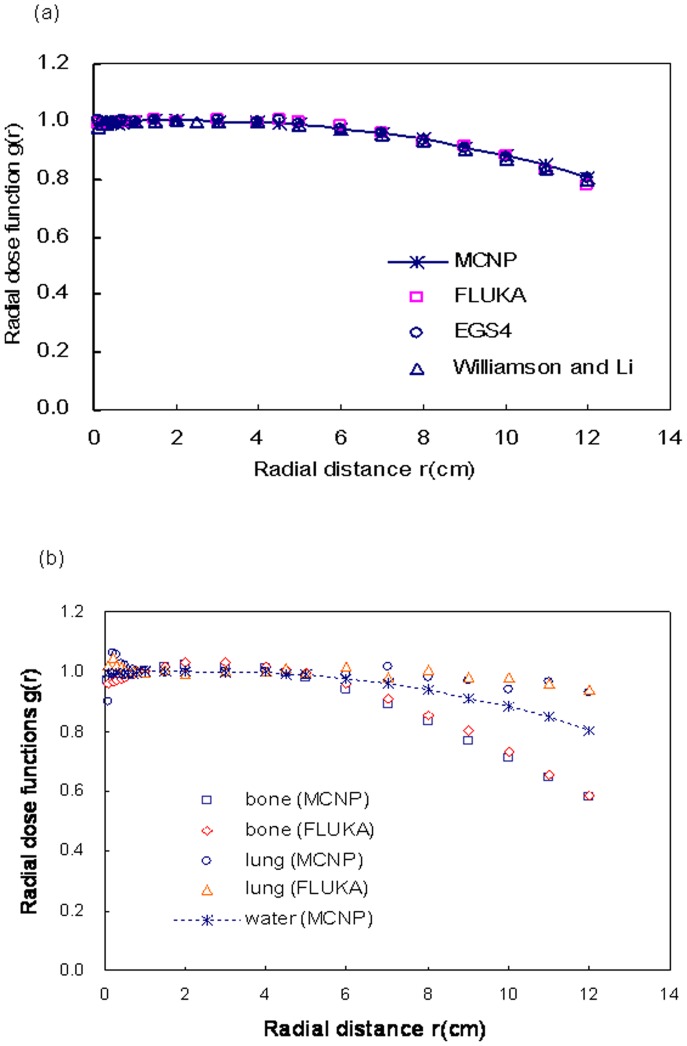
Radial dose function comparisons. Shown are simulation results for (a) comparison with Williamson and Li’s data and (b) radial dose functions in water, bone, and lung phantoms among the three Monte Carlo codes.

**Figure 4:**
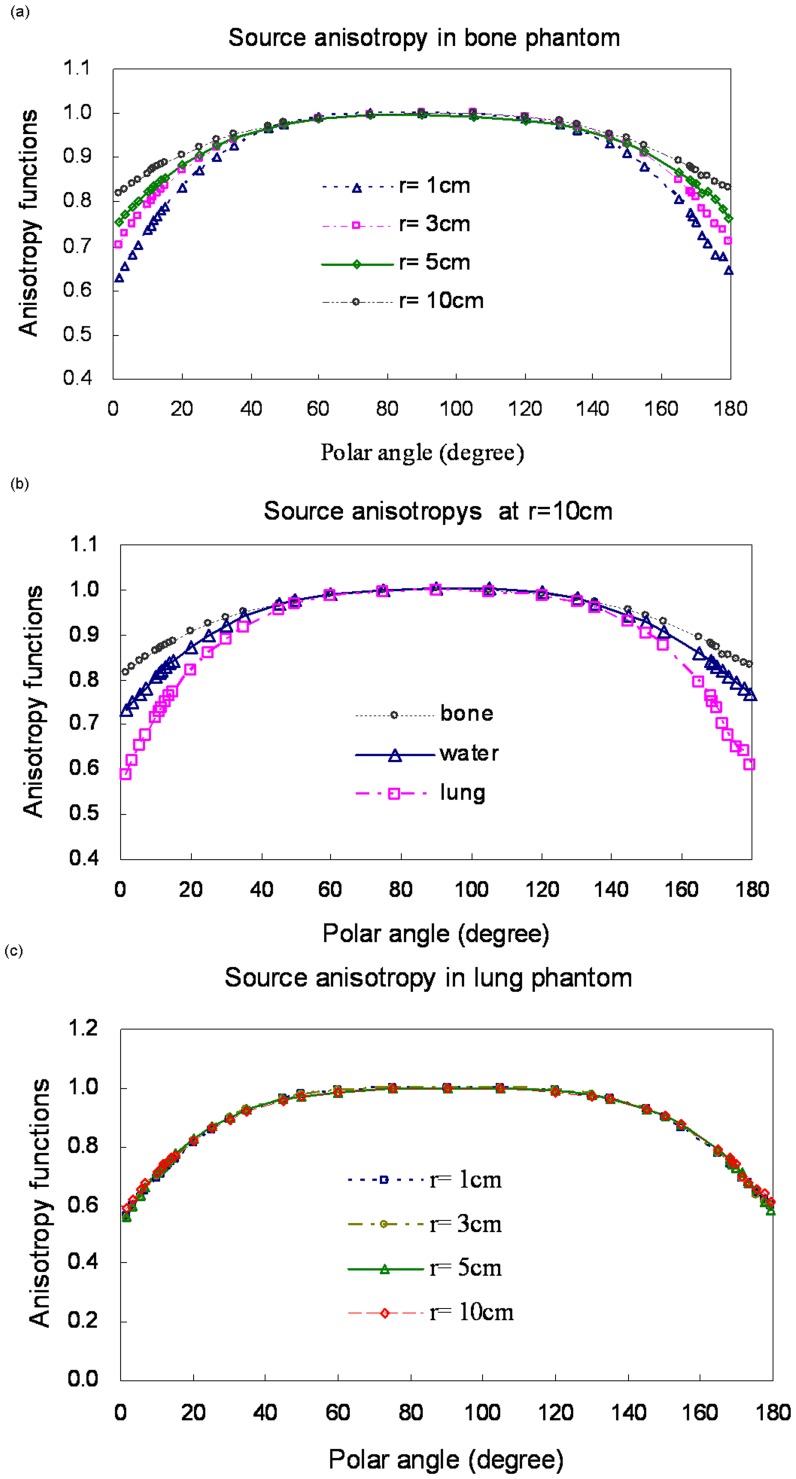
Anisotropy functions for different materials. (a) Bone at r = 1, 3, 5, and 10 cm; (b) water, bone, and lung at r = 10 cm; (c) lung at r = 1, 3, 5, and 10 cm.

**Figure 5:**
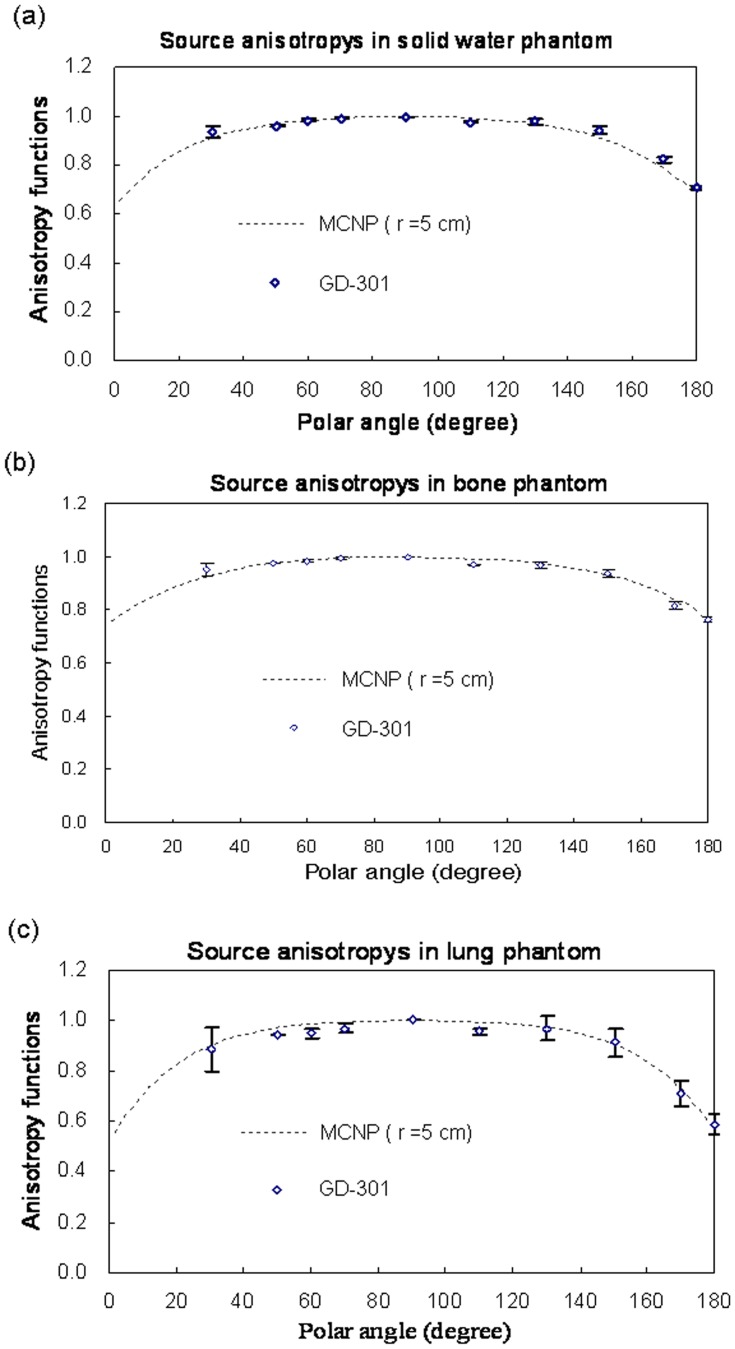
Comparisons of Monte Carlo anisotropy function simulation results with actual data for water, bone, and lung at r  = 5 cm from source center.

**Figure 6:**
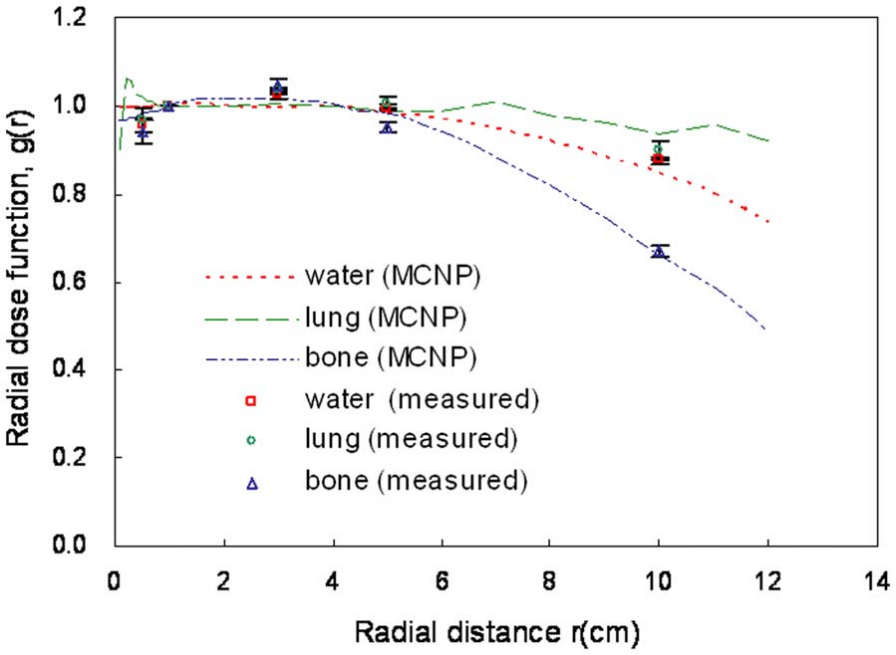
Comparison of Monte Carlo simulation results for radial dose function with GD-301 data for water, lung, and bone phantoms.

**Figure 7:**
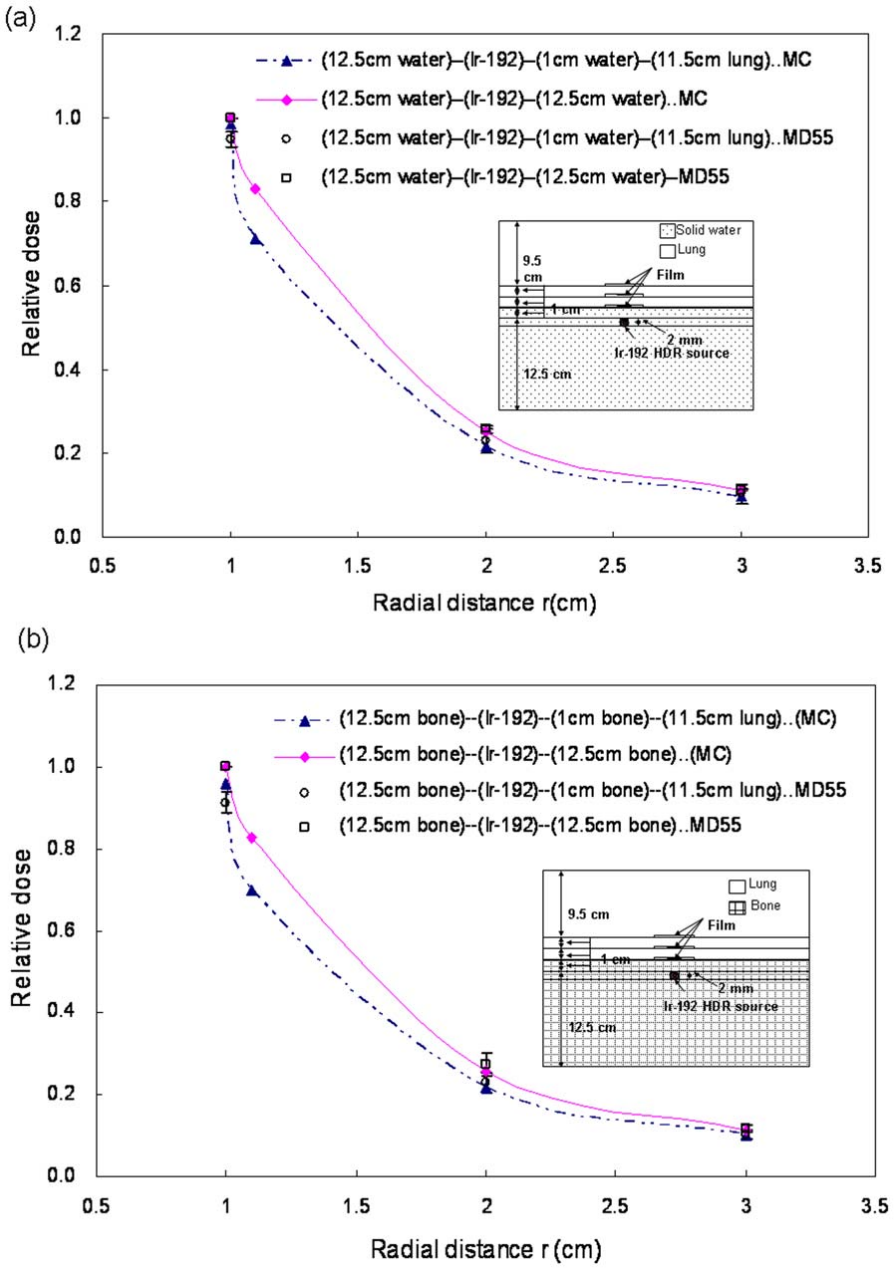
Comparison of interface doses between (a) solid water phantoms and solid water-lung interfaces, (b) homogeneous bone phantoms and bone-lung interfaces.

**Figure 8:**
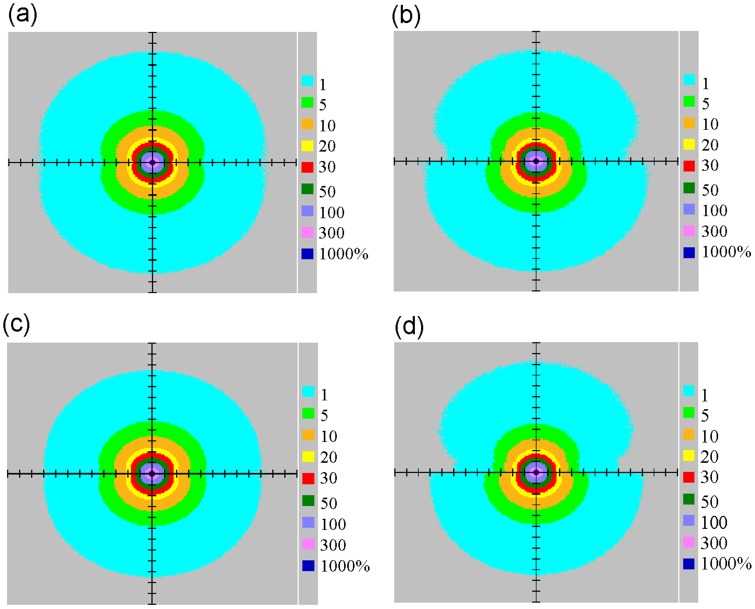
Isodose curves of (a) solid water, (b) solid water-lung interfaces, (c) bone, and (d) bone-lung interfaces.

## Materials and Methods

### Monte Carlo Simulations

In this study the ^192^Ir source (microSelectron HDR, Nucletron, Netherlands) used as an active source, 0.6 mm diameter ×3.5 mm long cylinder of pure iridium metal. Threshold energy photon and electron transport were set at 10 keV when performing our ^192^Ir dose calculations. In order to speed up calculations, particles were terminated when at least 10^8^ primary photons were detected outside of each phantom, yielding statistical errors below 3%. Each MC code contains detailed construction and identical ^192^Ir source data. In MC codes, the ^192^Ir source was positioned at the geometric center (origin point), thus allowing for comparisons with Williamson and Li’s calculations [11]. During particle transport, energy transferred between particles and phantoms are recorded in two-dimensional matrix representing the absorbed dose in a flat area in which the transverse axis of the source is located. Since the source is cylindrical and symmetrical to the transverse axis of the source, it was assumed that the 3-D dose distribution would appear to be symmetrical with the transverse axis at its center. Based on the TG-43 report, the major emission spectrum of photon for the ^192^Ir were 290 keV (14.34%), 308 keV (14.69%), 317 keV (40.96%), 468 keV (23.46%) and 608 keV (6.55%). On the other hand, the five energies listed from TG-43 were randomly determined by random number generator in MC code. Because the emission spectrum was taken from microSelectron HDR source model, the encapsulation material was not simulated in this study to avoid the doubly account of source term [14].

### Phantoms

We used solid water, bone, and lung phantoms for this research. The phantoms had the same geometric structures but vary in terms of material and density. Bone and lung phantom compositions were adopted from the International Commission on Radiation Units and Measurements Report 44 (ICRU 44). The two tissue types that had the largest density differences from water were cortical bone (1.92 g/cm^3^) and inflated lung (0.26 g/cm^3^).

### Radiophotoluminescent Glass Dosimeter Measurement

The RPLGD (model GD-301, Asahi Techno Glass Corporation, Shizuoka, Japan) used to verify our MC simulation results was calibrated using a ^60^Co treatment machine (model E-78, Atomic Energy of Canada Limited, Canada) Similar dose calibrations were performed for the Gafchromic film prior to making ^192^Ir-source dose measurements. RPLGD weight compositions were P (31.55%), O (51.16%), Al (6.12%), Na (11.00%), and Ag (0.17%) [15]. Effective RPLGD atomic number and density were 12.04 and 2.61 g/cm^3^ [16]. Dose readouts were performed with a FGD-1000 reader (Asahi Techno Glass). When set to the high-dose range, effective GD-301 dosimeter readout size was 1 mm in diameter and 0.6 mm deep [16], [17], making the GD-301 ideal for brachytherapy dose measurements [18].

To support Monte Carlo simulation results, radial and anisotropy measurements were performed in phantom using glass dosimeters. The GD-301 glass dosimeters were repeatedly given 15 Gy by ^60^Co treatment machine to study the readout reproducibility. Identical measurement methods were used for the solid water, bone, and lung tissue phantoms. Slabs were sandwiched to build 25×25×25 cm^3^ phantoms; dual radii and multi-angle directional measurement points were simultaneously measured for each side (Figure 1).

The large dose gradient around brachytherapy sources necessitated the use of a small detector with a wide dose detection range. Gafchromic film (MD-55, Nuclear Associates, USA) was used due to its excellent spatial resolution (0.2 mm), tissue equivalent 6.5 atomic number, and low sensitivity to room luminosity [19], [20], [21]. The MD-55 has a thickness of approximately 0.3 mm and dose ranges from 3 to 100 Gy. Measurements were performed for solid water, solid water to lung, homogeneous bone, and bone to lung (Figure 2). Two similar phantoms were used to measure the interface dose between solid water-lung and bone-lung (also shown in Figure 2 with identical film placement process). A Kodak LS50 film digitizer was used to scan all MD-55 films, calibrations, and experiments 2-day post-irradiation.

### Physical Characteristics of the Dosimeters

In this study, physical characteristics of the GD-301 and the MD-55 were examined. These included identification of the dose linearity, energy dependence, and angular dependence. At each point, five dosimeters were used to verify the linearity between the signals and the received radiation dose ranging from 3 Gy to 120 Gy. Since ^60^Co was used as the calibration source in this study, this experiment also explored the energy response of GD-301 and MD-55 for both ^192^Ir and ^60^Co. This study chose eight points within the energy range from 22 to 1250 keV, including 22, 25, 38, 67, 102, 151, 662 (^137^Cs) and 1250 keV (^60^Co). The relative response of each dosimeter was normalized to the ^137^Cs readout value. For angular dependence study, the ^60^Co was used to deliver 15 Gy for dosimeters with 11 different entrance angles, including 0°, ±10°, ±30°, ±60°, ±70° and ±80°. The relative response of each dosimeter was normalized to the 0° readout value.

## Results and Discussion

### Physical Characteristics of the Dosimeters

R^2^ is the proportions of variability in the data set that is accounted for by the statistical model. As R^2^ approached 1, the relationship between readout value and irradiation dose is a direct proportional function. R^2^ of GD-301 and MD-55 were 0.9995 and 0.9984, respectively. When the relative response was normalized to 662 keV, the energy dependence of MD-55 was decreased by 18% at 22 keV. Since the effective atomic number was 12.04 for GD-301, the energy dependence of GD-301 reached 320% at 38 keV. Radiation sensitivity of MD-55 was not significantly affected by entrance angles in the range of 0° to ±80°. When the entrance angle was - 80°, the GD-301 response was 7% lower than that at 0°. This data can be explained by the radiophotoluminescence signal readout center of GD-301, which is 0.7 mm away from its edge and under such circumstance could result in radiation attenuation [18].

### Dose Distribution and Radial Dose Function Comparison

All emission particles for our MC calculations were produced by the source, and statistical errors decreased closer to the source. Statistical performance would be less optimal if calculations were made farther away, due to the lower number of active particles per unit volume. At least 10^8^ particles were emitted for each MC simulation, thereby keeping standard errors below 3% at a distance of 12 cm from the source in each phantom.

The radial dose function, g(r), accounts for the effects of absorption and scatter in the medium along the transverse axis of the source [1]. Dose rate in water were radially calculated 1 cm from the source, thus indicating changes in the relative dose values of phantom along the radial direction. According to the inverse square law, the dose at 0.5 cm was approximately 4 times that at 1 cm, and the dose at 2 cm was approximately 1/4 that at 1 cm. Our *g(r)* calculations at *r* radial distances from 0.1 to 12 cm are shown in Figure 3. These simulation results agree with Williamson and Li’s calculations (<1.7% difference) [11]. As shown in Figure 3b, as depth increased (a) the radial dose function in bone decreased much more than in water (due to the higher attenuation coefficient in bone) and (b) radial dose function decreased less in lung tissue than in water.

### Anisotropy Function Comparison

The anisotropy function accounts for the anisotropy of dose distribution around the source, including the effects of absorption and scatter in the medium [1]. In bone, the anisotropy function becomes less pronounced with increasing distance from the source. In contrast, the anisotropy function becomes more pronounced in lung. Our MCNP4C code results match those reported by Williamson and Li [11]. As shown in Figures 4a and b, *F(r,θ)* in bone resembles a point source because the scattered rays are at greater distances from the source in water (gradually decreasing differences in all directions). As shown in Figure 4c, a distinct *F(r,*θ*)* differences were not noted in the lung phantom. These results are similar to the air measurements reported by Muller-Runkel and Cho [7].

### Radiophotoluminescent Glass Dosimeter Measurements

In the absence of other dose parameter descriptions for bone and lung phantoms, we designed an experiment to collect point dose measurements to verify our MC results. Note that the GD-301 readout size is small, has better spatial resolution, and therefore is less affected by dose gradients. Our data represent average values from three measurements. We compared our MCNP4C calculations for anisotropy functions with GD-301 measurement results at a radius of 5 cm (Figure 5). Our MC calculations statistical error was within 3%. Comparisons of the MCNP4C results with radial dose function measurements in phantoms for the three different materials are shown in Figure 6.

### Gafchromic Film Measurements

Dose was calculated using MCNP4C codes, normalized to homogeneous phantom doses at *r* of 1 cm. Differences between solid water-lung interface and solid water ranged from 0.6% to 14.4% (Figure 7a), and between bone-lung interface and bone ranged from 4.1% to 15.7% (Figure 7b). According to the isodose curves, we found that the dose for water-lung interface was less than solid water, while the dose for bone-lung interface was less than bone (Figure 8). Muller-Runkel and Cho pointed out that the denser medium can induce a larger scatter [7]. Therefore, we concluded that the scatter component had different magnitudes due to different material densities.

### Conclusion

In this study we used three Monte Carlo codes (EGS4, FLUKA and MCNP4C) to calculate ^192^Ir dose distributions in water, bone, and lung phantoms and their interfaces for comparison with water phantom data reported by Williamson and Li [11]. No material was available for comparisons with calculated results for bone and lung phantoms. According to the parameters of TG-43, the geometry factor accounts for the variation of relative dose due to spatial distribution of activity within the source, but neglect photon absorption and scattering in the source structure. Radial dose and anisotropy functions were dependent on different phantom materials. Our results indicate a direct relationship between increased dose variance and increased material density due to the different magnitudes of scatter components. Good correlation was found between our film data and the calculated results.

Our conclusion is that clinical parameters, followed TG 43, used in HDR treatment planning system do not satisfy the dose accuracy for different tissue materials. Therefore, we suggest the dose calculation algorism used in HDR should take into account for different tissue density to improve the dose accuracy. Furthermore, it should take into account the interface dosimetry between tissues with different densities. We hope this data will support further efforts to understand dose distribution differences in water, bone, and lung tissue, as well as their interfaces. The calculation of TPS is based on the TG-43 calculation algorithm. Indeed, the better dose calculation may not improve overall treatment quality at the beginning because people have to build new experiences.
